# Primary Squamous Cell Carcinoma of the Pancreas as a Cause of Biliary Obstruction

**DOI:** 10.7759/cureus.856

**Published:** 2016-10-31

**Authors:** Kyle Rowe, Jeet Mehta, Fredy Nehme, William Salyers

**Affiliations:** 1 Internal Medicine, University of Kansas School of Medicine - Wichita; 2 Pediatrics, University of Kansas School of Medicine - Wichita

**Keywords:** jaundice, scc (squamous cell cancer), pancreatic cancer, endoscopic ultrasound, squamous cell carcinoma of the pancreas, biliary obstruction

## Abstract

Primary squamous cell carcinoma of the pancreas (SCCP) is a rare neoplasm, given a lack of naturally occurring squamous cells within the pancreas, accounting for only 0.2% of all pancreatic cancers. The etiology is unknown. Symptomatology is non-specific and similar to other pancreatic neoplasms. No non-invasive testing can adequately rule in SCCP, and workup should proceed similarly to any pancreatic mass. Tissue sampling is required for diagnosis and guidance of further management, most commonly by endoscopic ultrasound with fine needle aspirate. SCCP is more aggressive than adenocarcinoma of the pancreas with a median survival of three and ten months for those treated with palliative and surgical intent, respectively. The optimal treatment regimen remains unknown, though the uses of radiation therapy, platinum-based regimens, gemcitabine, and 5-FU have all been reported with favorable results. We present a case of primary SCCP in an 81-year-old female who presented with jaundice.

## Introduction

Primary squamous cell carcinoma of the pancreas (SCCP) is a rare neoplasm with an unknown etiology. A 2016 SEER database review by Makarova-Rusher et al., from 2000 to 2012, found only 214 cases, accounting for 0.2% of all pancreatic cancers [1]. This corresponds to an annual incidence rate of approximately 0.02 cases per 100,000. For perspective, the annual incidence rate of pancreatic adenocarcinoma is approximately 6.9 cases per 100,000 [1]. Squamous cells do not naturally occur in the pancreas, though squamous metaplasia has been noted in the setting of chronic pancreatitis and after the placement of pancreatic duct stents [2]. Squamous cells have also been noted to line certain non-malignant pancreatic cysts [3]. Though squamous cells have been noted in these circumstances, the true progenitors to SCCP are not known. SCCP should be differentiated from adenosquamous carcinoma of the pancreas, which has been reported with a higher incidence of 0.5 cases per 100,000, and unlike SCCP, adenosquamous carcinoma displays genetic markers of ductal origin [4]. Alternative sources for primary squamous malignancy must always be ruled out as metastatic lesions to the pancreas are not uncommon and have been reported from squamous esophageal and lung cancer [5-6]. We present a case of a primary SCCP in an elderly female who presented with jaundice.

## Case presentation

**Figure 1:**
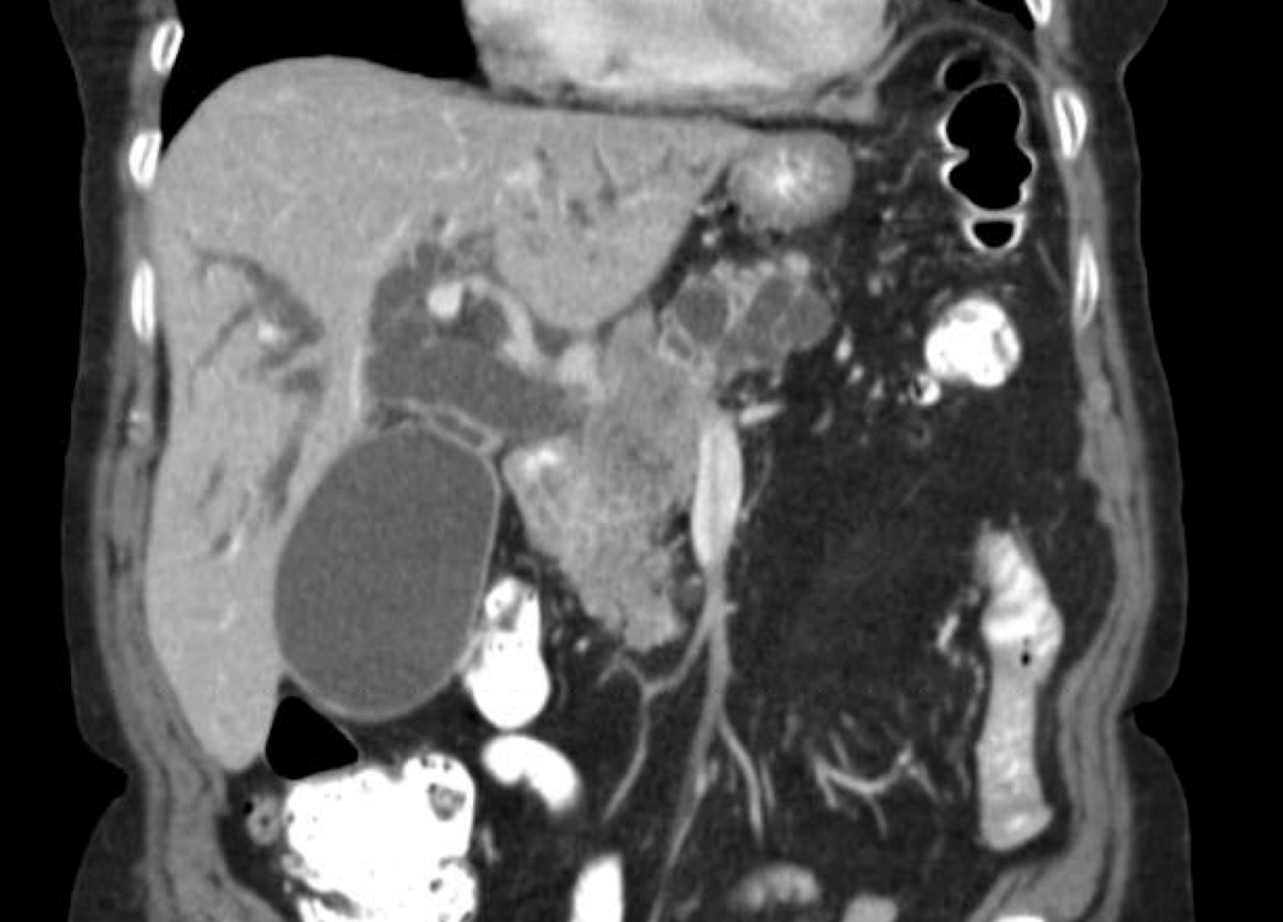
CT Abdomen with Contrast CT scan with contrast demonstrating large pancreatic head mass causing biliary obstruction with extensive intrahepatic and extrahepatic ductal dilatation and a distended gall bladder.

An 81-year old female with a past medical history significant for hypertension, hypothyroidism, and osteoporosis presented for acute onset of painless jaundice. Laboratory testing revealed an elevated total bilirubin level of 3.9 mg/dL with elevated transaminases (ALT 243, AST 240). Computed tomography (CT) scan of the abdomen and pelvis showed a large pancreatic mass measuring 4.8 x 3.3 cm, diffuse dilatation of both intrahepatic and extrahepatic biliary systems, and a large distended gallbladder (Figure 1).

**Figure 2:**
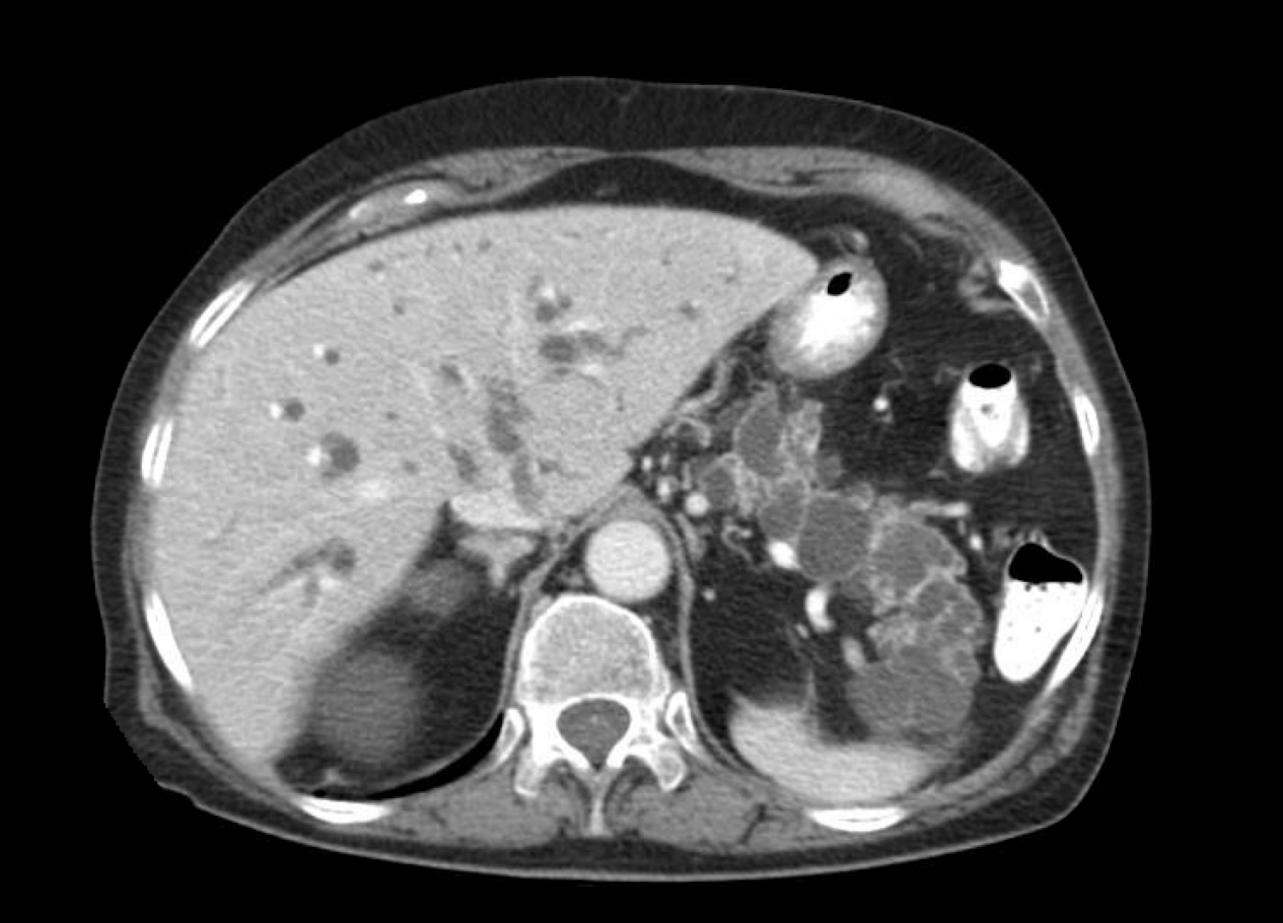
CT Abdomen with Contrast Contrast CT scan demonstrating numerous secondary cystic dilatations in the tail of the pancreas.

The pancreatic mass encroached on the celiac axis and encased the portal vein, but no definitive evidence of vascular invasion was reported on CT scan. The tail of the pancreas was noted to have numerous secondary cystic dilatations (Figure 2).

**Figure 3:**
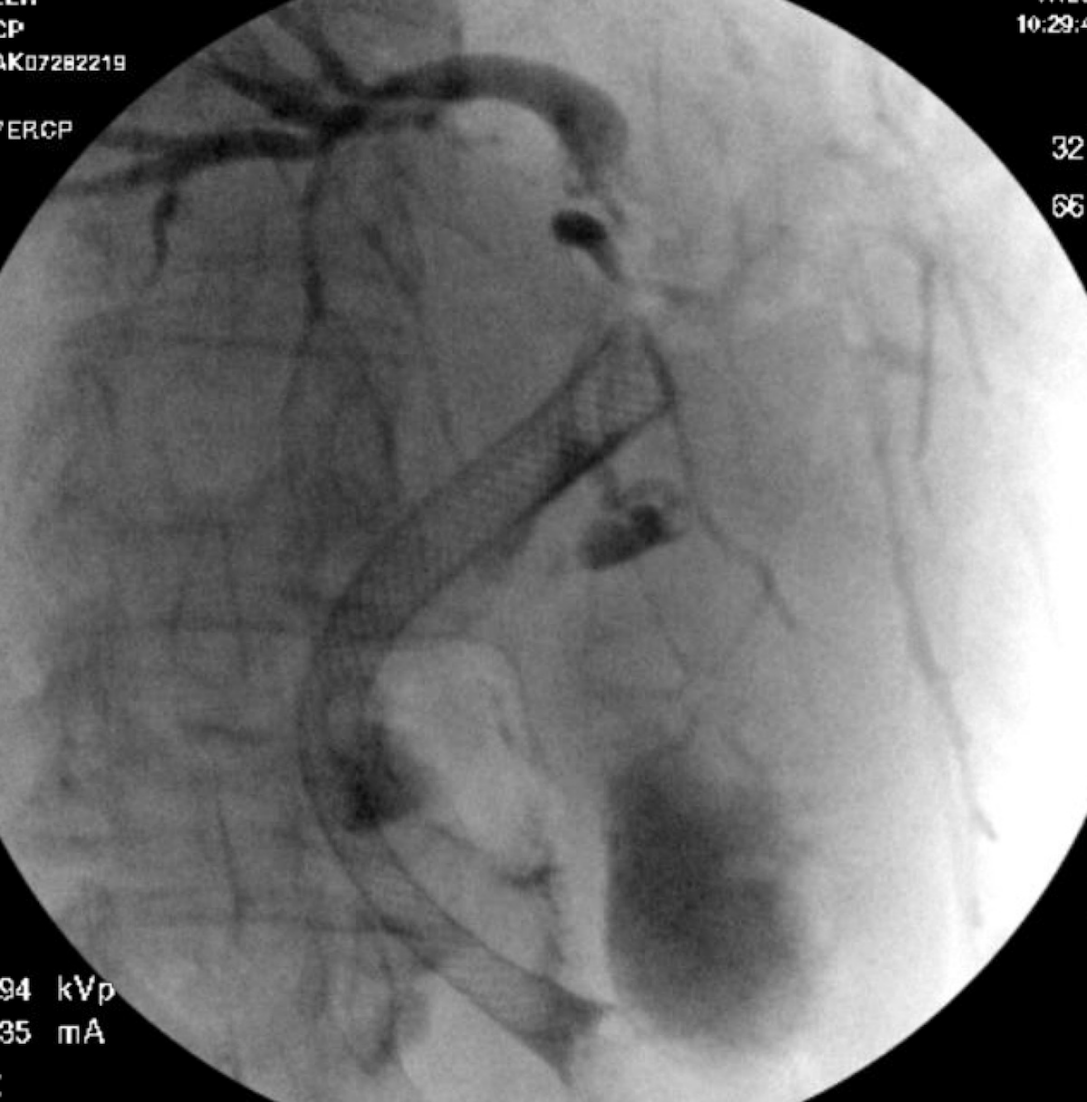
ERCP Fluoroscopy Images obtained during ERCP demonstrating stenting of the common bile duct with adequate drainage into the small bowel.

A percutaneous biliary drain was successfully placed. Endoscopic ultrasound (EUS) revealed a 52.8 x 44.6 mm mass in the head and neck of the pancreas and demonstrated invasion into the portal vein and celiac axis. No celiac lymphadenopathy was identified. A fine needle aspiration (FNA) of the mass was performed, and samples were sent for cytopathology. Next, an endoscopic retrograde cholangiopancreatogram (ERCP) with sphincterotomy was performed, and a fully covered metal biliary stent was placed traversing the mass-induced stricture in the distal common bile duct. Subsequent imaging demonstrated adequate internal drainage of the biliary tree (Figure 3).

**Figure 4:**
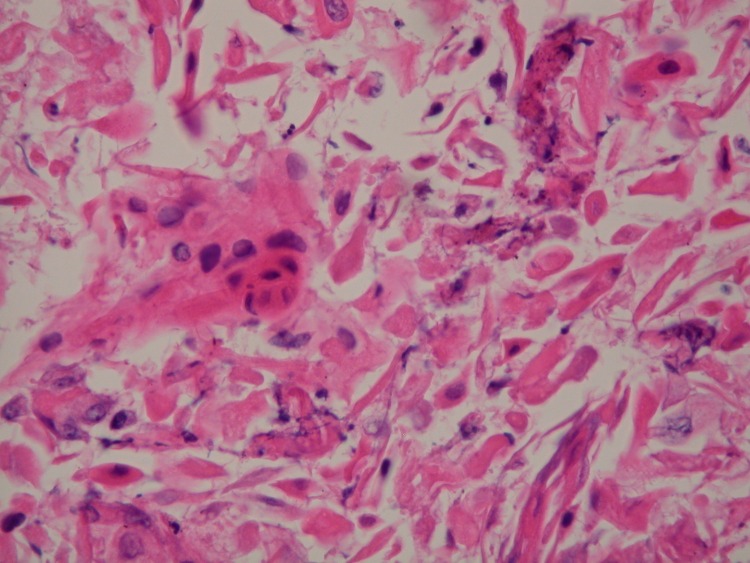
Pancreatic FNA Cytology H&E stained cytology from a pancreatic fine needle aspirate demonstrating dysplastic squamous cells.

Cytopathology revealed keratinizing squamous cell carcinoma (Figure 4).

The patient agreed to participate and was explained the nature and objectives of this study, and informed consent was formally obtained. No reference to the patient's identity was made at any stage during data analysis or in the report.

A workup for an alternative primary tumor was negative, including negative esophagogastroduodenoscopy for esophageal mass (performed during EUS), and CT scan of the chest, abdomen, and pelvis. CEA level was normal at 3.8 mcg/L and CA 19-9 was slightly elevated at 44 U/ml. PET-CT showed a large, hypermetabolic pancreatic head mass compatible with primary neoplasm. The liver showed multiple hypermetabolic lesions, with a lesion near the porta hepatis having a standardized uptake value (SUV) max of 8.5. The large mass in the head of the pancreas had an SUV max of 18.5. No hypermetabolic lymph nodes were identified in the retroperitoneum. A diagnosis of metastatic primary squamous cell carcinoma of the pancreas (SCCP) was made. Given her age, decreased performance status, and evidence of vascular invasion of the mass, her primary goal of care was to proceed with palliative therapy.

## Discussion

The majority of patients with SCCP lesions present with jaundice, though non-specific constitutional symptoms including weight loss, malaise, and abdominal pain are common [7]. Non-invasive testing cannot adequately differentiate SCCP from other pancreatic neoplasms, and the workup proceeds similarly to any pancreatic mass. Most patients will undergo initial imaging by a CT scan. Iodinated contrast has been reported to result in higher attenuation in SCCP compared to adenocarcinoma, though this has not been reproduced [8]. EUS with FNA is recommended as a next step in diagnosis and staging a new solitary pancreatic mass, especially when resectability is difficult to determine by primary imaging. This case highlights one example of increased sensitivity of EUS to detect vascular invasion over CT, though studies regarding this have been mixed [9]. In cases of non-resectable disease, tissue diagnosis by FNA is still required to guide the management of palliative radiation/chemotherapy. In the case of SCCP, up to 56% of cases were noted to be metastatic at presentation, with 61% being Stage T3-T4 [1].

The initial approach of treatment for SCCP is similar to that of adenocarcinoma, where resection results in improved mortality in cases of non-metastatic disease without vascular involvement. Makarova-Rusher et al., reported that median survival for patients with SCCP treated with palliative versus surgical intent was 3 and 10 months respectively, compared to 5 and 18 months for those with adenocarcinoma [1]. In cases of non-resectable disease, the optimal palliative regimen is unknown. The uses of radiation therapy, platinum-based regimens, gemcitabine, and 5-FU have all been reported with favorable results, though the overall mortality remains high [7, 10]. Further research is needed in this area.

## Conclusions

SCCP is a rare and aggressive pancreatic malignancy. Diagnosis proceeds similarly to other pancreatic masses, and EUS with FNA has been proven to be useful in guiding further management. Surgical treatment results in improved mortality, though the optimal palliative regimen remains unknown. Often locally advanced or metastatic-at-presentation, mortality is higher in SCCP than that of the more common adenocarcinoma. Our case highlights a primary SCCP that should alert clinicians about the rarity of the diagnosis.
